# Effects of GSK3 inhibitors on in vitro expansion and differentiation of human adipose-derived stem cells into adipocytes

**DOI:** 10.1186/1471-2121-9-11

**Published:** 2008-02-13

**Authors:** Laure-Emmanuelle Zaragosi, Brigitte Wdziekonski, Coralie Fontaine, Phi Villageois, Pascal Peraldi, Christian Dani

**Affiliations:** 1 Institute of Signalling, Biology of Development and Cancer, University of Nice Sophia- Antipolis, CNRS 6543, 28 avenue Valrose, 06100 Nice, France

## Abstract

**Background:**

Multipotent stem cells exist within adipose tissue throughout life. An abnormal recruitment of these adipose precursor cells could participate to hyperplasia of adipose tissue observed in severe obesity or to hypoplasia of adipose tissue observed in lipodystrophy. Therefore, pharmacological molecules that control the pool of stem cells in adipose tissue are of great interest. Glycogen Synthase Kinase (GSK) 3 has been previously described as involved in differentiation of preadipose cells and might be a potential therapeutic target to modulate proliferation and differentiation of adipocyte precursors. However, the impact of GSK3 inhibition on human adipose-derived stem cells remained to be investigated. The aim of this study was to investigate GSK3 as a possible target for pharmacological inhibition of stem cell adipogenesis. To reach this goal, we studied the effects of pharmacological inhibitors of GSK3, i.e. lithium chloride (LiCl) and BIO on proliferation and adipocyte differentiation of multipotent stem cells derived from human adipose tissue.

**Results:**

Our results showed that GSK3 inhibitors inhibited proliferation and clonogenicity of human stem cells, strongly suggesting that GSK3 inhibitors could be potent regulators of the pool of adipocyte precursors in adipose tissue. The impact of GSK3 inhibition on differentiation of hMADS cells was also investigated. Adipogenic and osteogenic differentiations were inhibited upon hMADS treatment with BIO. Whereas a chronic treatment was required to inhibit osteogenesis, a treatment that was strictly restricted to the early step of differentiation was sufficient to inhibit adipogenesis.

**Conclusion:**

These results demonstrated the feasibility of a pharmacological approach to regulate adipose-derived stem cell function and that GSK3 could represent a potential target for controlling adipocyte precursor pool under conditions where fat tissue formation is impaired.

## Background

Obesity, which is characterized by an excess of adipose mass, is a major public health-problem. Hypertrophy, i.e. increase in the adipocyte size and hyperplasia, i.e. increase in the adipocyte numbers, are observed in severe obesity. It is now well established that multipotent stem cells exist within adipose tissue throughout the life [1-3] and that an excessive recruitment of these adipose precursor cells could lead to hyperplasia. As opposed to hypertrophy, hypoplasia of adipose tissue is observed in lipodystrophy and is associated with diabetes and hyperlipidaemia. Adipocytes and osteoblasts share the same mesenchymal precursor cell [4]. Adipogenesis and osteogenesis are processes that respond to a balance in bone marrow and this balance can be disrupted under pathological conditions such as osteoporosis where adipocytes develop at the expense of osteoblasts [5]. Therefore, pharmacological molecules that control the pool of adipose stem cells are of great interest.

Glycogen synthase kinase 3 (GSK3), a serine/threonine kinase existing in two isoforms GSK3α and GSK3β, is a key regulator of numerous signalling pathways. In particular, GSK3 has been involved in multiple cellular processes including Wnt and Hedgehog (Hh) pathways. In the activation of the canonical Wnt pathway, inhibition of GSK3 results in dephosphorylation of β-catenin leading to its nuclear accumulation. Inhibition of GSK3 also contributes to activation of the Hh pathway by stabilisation of Gli 2/3 transcription factors, favouring their nuclear translocation and leading to transcription of target genes. Gli1 is one of them and induction of Gli1 gene expression has been characterized as a reliable marker of Hh signalling activity [6]. The role of GSK3 in the differentiation of preadipose cells has been previously described. It has been shown that activation of the Wnt pathway via inhibition of GSK3 inhibits adipogenesis of murine preadipocytes and in mice [7,8]. Expression of Hh target genes was reduced in fat depots of obese mice, suggesting anti-adipogenic properties of this pathway [9]. GSK3 is also a key component of the circadian apparatus. The circadian clock may play a role in adipocyte metabolism and it has been recently shown that inhibition of GSK3 in human adipocytes lengthened the period of expression of core circadian transcriptional apparatus [10]. Therefore, GSK3 could represent a potential therapeutic target to modulate proliferation and differentiation of adipocyte precursors. However, the impact of GSK3 inhibition on human adipose-derived stem cells remained to be investigated. To address this point we have studied the effects of two pharmacological inhibitors of GSK3, lithium chloride (LiCl) [11] and 6-bromoindirubin-3'-oxime (BIO) [12], on multipotent stem cells derived form human adipose tissue (hMADS cells, also named ASC as suggested by IFATS, a society focusing on Adipose-derived Stem or Stromal Cells, and discussed by Mitchell et al. [13]. We have previously established the procedure to isolate and expand hMADS cells from different donors. hMADS cells exhibit key features of mesenchymal stem cells such as self-renewal capacity and ability to undergo differentiation at the single cell level into at least two lineages (adipogenic and osteogenic) [14,15]. Thus, hMADS cells represent a potent cellular model to investigate pathways regulating self-renewal, adipogenesis and osteogenesis [16,17].

## Results and Discussion

### Functional inhibition of GSK3 in human adipose-derived stem cells

**Figure 1:**
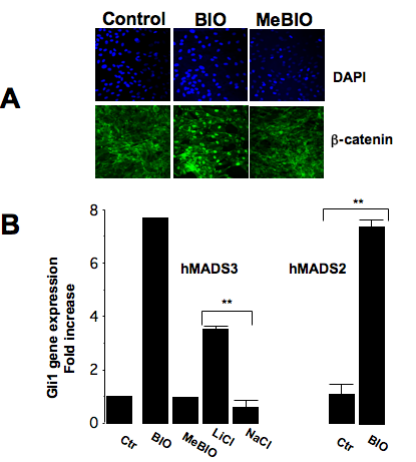
**GSK3 inhibitors induced β-catenin nuclear translocation and Gli1 gene expression**. hMADS3 cells were maintained in proliferation medium supplemented with 0.5% FCS, then localisation of β-catenin was revealed after treatment with 0.5 μM BIO or 0.5 μM MeBIO for 24 hours. DAPI was used to label nuclei (A). hMADS2 and hMADS3 cells were maintained as in (A) and fold induction in the expression of Gli1 gene was quantified by real-time PCR 24 hours after treatment with 0.5 μM BIO, 0.5 μM MeBio, 20 mM LiCl or 20 mM NaCl or under control condition (Ctr). Bars are the means ± S.E of 2 independent experiments, **: p < 0.01 (B).

In order to determine the effective concentrations of BIO and LiCl, nuclear translocation of β-catenin and induction of Gli1 gene expression have been analyzed in hMADS2 and hMADS3 cells, two stem cell populations isolated from separate donors. As shown in Fig 1A, stimulation of hMADS3 cells with 0.5 μM BIO led to nuclear accumulation of β-catenin, whereas the inactive BIO molecule (MeBio [12]) had no effect. Under our culture conditions, 0.1 μM BIO had no effect on β-catenin stabilization and 5 μM led to cell death (not shown). Treatment of cells with 20 mM LiCl had the same effect than 0.5 μM BIO whereas 10 mM LiCl and 20 mM sodium chloride (NaCl) had no effect (not shown). Nucleofection of hMADS3 cells with a reporter gene under the control of Tcf/Lef response-element showed that the β-catenin pathway was activated in hMADS cells after inhibiting GSK3 (see Additional file 1). Gli1 gene expression was also stimulated in hMADS3 and hMADS2 cells that were maintained in the presence of 0.5 μM BIO or 20 mM LiCl (Fig. 1B).

### Effect of GSK3 inhibitors on proliferation of human adipose-derived stem cells

**Figure 2:**
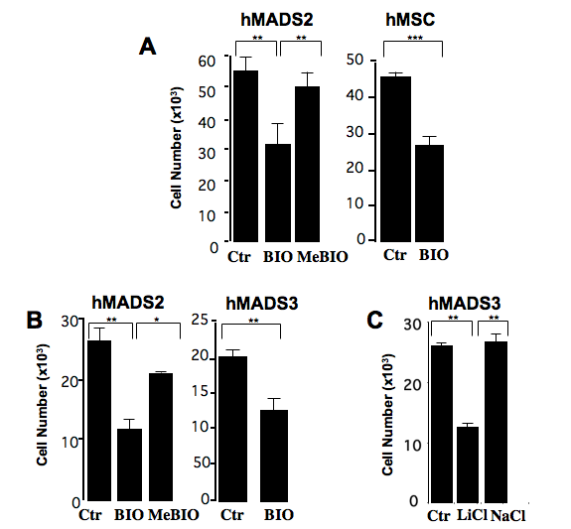
**Impact of GSK3 inhibition on the proliferation of hMADS cells**. Cell number after treatment of hMADS3 cells for 5 days (A) or 3 days (B, C) with indicated compounds. hMADS cells were maintained in 0.5% FCS whereas hMSCs were maintained in medium supplemented with 10% FCS. Bars are the means ± SE of 3 independent experiments. *: p < 0.05, **: p <0.01, ***: p < 0.001

**Figure 3:**
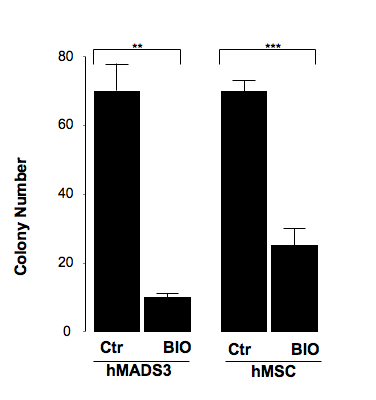
**Effects of BIO on hMADS3 cell clonogenicity**. hMADS cells and hMSCs were plated at clonal density and maintained in medium supplemented with 10% FCS (Ctr). One day after cell plating, 0.5 μM BIO was added to the culture medium. Fifteen days later, the number of colonies was scored. Bars represent means of 2 independent experiments. **: p < 0.01, ***: p < 0.001

Then, the functional consequence of GSK3 inhibition on proliferation of hMADS cells has been investigated. Morphology of hMADS cells changed after treatment with 0.5 μM BIO or 20 mM LiCl. From a fibroblastic-like morphology, GSK3 inhibitor-treated cells displayed a cuboidal shape (see Additional file 2). We did not observe any change in morphology or any effect on proliferation when cells were treated with 0.1 μM BIO (not shown). In contrast, at a higher concentration of 0.5 μM BIO, treatment of hMADS2 cells for 5 days led to a 40–50% inhibition of proliferation. Effects of BIO on cell morphology and proliferation were reversible indicating that the GSK3 inhibitor did not affect cell viability (not shown). MeBio-treated cells did not display any significant inhibition of proliferation. The inhibitory effect of BIO was also observed after treatment of mesenchymal stem cells isolated from human bone-marrow (hMSCs) (Fig. 2A). BIO-inhibitory effect was detected as early as 3 days after treatment and was reproducible on hMADS3 cells (Fig. 2B). Inhibition on hMADS3 cell proliferation was also observed to a similar extent after treatment with 20 mM LiCl but not with NaCl (Fig. 2C). As shown in Fig. 3, BIO inhibited the ability of hMADS cells and hMSCs to proliferate at the single cell level, a crucial feature of stem cells. This indicates that BIO cannot be used to expand mesenchymal stem cells *ex vivo*. This is in contrast with a previous report showing that BIO was able to maintain pluripotent embryonic stem cells by activating Wnt pathway [18] and that inhibition of β-catenin promotes proliferation of uncharacterized stromal cells of adipose tissue [19]. These discrepancies could be due to a differential effect of Wnt pathway in embryonic versus human adult mesenchymal stem cells or/and to the fact that, as shown above, BIO activated both Wnt and Hh pathways within the same cell population. The effect of Hh pathway on proliferation on hMADS cells remains to be investigated. In addition, as also shown above, GSK3 is involved in several pathways and we cannot rule out that BIO could have other targets than GSK3 in hMADS cells. Altogether, these data show that BIO inhibited the proliferation of human stem cells, strongly suggesting that GSK3 inhibitors could be potent regulators of the pool of adipocyte precursors in adipose tissue.

### Effects of BIO on differentiation of human adipose-derived stem cells

To investigate the effect of GSK3 inhibition on adipocyte differentiation, hMADS cells were induced to differentiate in the presence of BIO or MeBIO. Adipocytes and osteoblasts share the same mesenchymal precursor cell. Adipogenesis and osteogenesis are processes that respond to a balance in bone marrow and this balance can be disrupted under pathological conditions such as osteoporosis in which adipocytes develop at the expense of osteoblasts [5,20]. We took advantage of hMADS cells that were previously demonstrated to differentiate in vitro into functional adipocytes and osteoblasts [16,17], to investigate the effect of GSK3 inhibition on both lineages.

**Figure 4:**
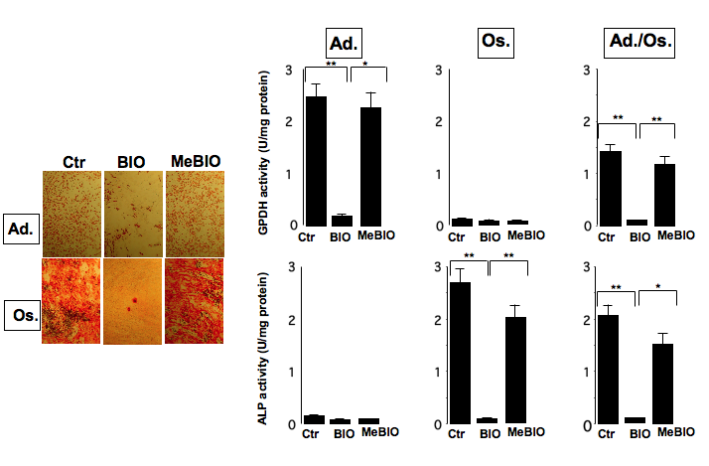
**Effects of BIO on the adipogenic and osteogenic potential of hMADS cells**. hMADS3 cells were induced to undergo differentiation into adipocytes (Ad.) or osteoblasts (Os) or both (Ad./Os.) in the absence (Ctr) or presence of 0.5 μM BIO or 0.5 μM MeBIO. Ten days later, cells were stained with Oil Red O for adipocytes or with Alizarin red for osteoblasts (left panel). Effects of the compounds on GPDH, expressed in adipocytes only, and ALP, expressed in osteoblasts only, enzymatic activities are shown (right panel). Bars are the means ± SE of 3 independent experiments. *: p < 0.05, **: p < 0.01

Adipogenic and osteogenic differentiations, as indicated by Oil Red O and Alizarin staining respectively, were dramatically inhibited in the presence of BIO, whereas MeBIO did not display any significant effect (Fig. 4, left panel). BIO-inhibitory effect was quantified using enzymatic activities such as glycerol-3-dehydrogenase (GPDH), which is expressed in adipocytes but not in osteoblasts, and alkaline phosphatase (ALP) activity, which is expressed in osteoblasts but not in adipocytes. As shown in Fig. 4, GPDH activity was inhibited when hMADS cells were maintained for 10 days in adipogenic medium or in a medium allowing simultaneously adipogenic and osteogenic differentiations supplemented with BIO. ALP activity was also inhibited when cells were maintained in osteogenic or adipogenic/osteogenic media supplemented with the GSK3 inhibitor (Fig. 4, right panel). Then, we investigated the impact of BIO treatment during cell proliferation on subsequent differentiation. Quantification of GPDH enzymatic activity indicated that cells previously treated with BIO during proliferation displayed a lower capacity to undergo adipocyte differentiation than untreated- or MeBio-treated cells (see Additional file 3). This result indicates that GSK3 plays a role in maintaining the differentiation potential of undifferentiated hMADS cells. This is reminiscent of our previous work showing the critical role of FGF2 pathway on self-renewal of hMADS cells [14].

**Figure 5:**
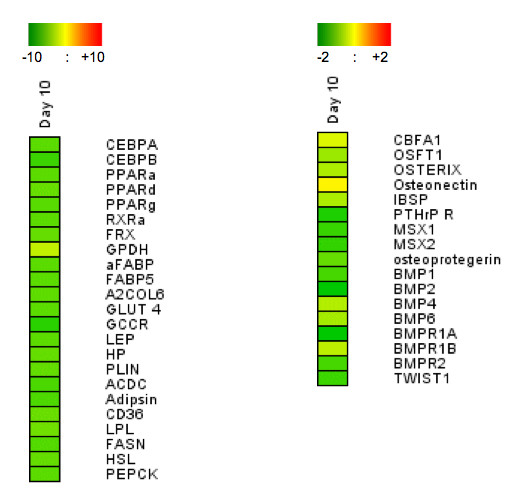
**Effects of BIO on expression of adipogenic and osteogenic genes**. hMADS3 cells were induced to undergo differentiation into adipocytes (left panel) or osteoblasts (right panel) in the presence of 0.5 μM BIO or 0.5 μM MeBIO. Ten days later, RNAs were prepared and expression of adipogenic or osteogenic markers was checked by qPCR. Results are the log2 inductions of BIO treated cells versus MeBIO treated cells. Expression data are colour-coded according to the scale.

Finally, analysis of adipocyte- and osteoblast-gene expression confirmed that treatment of hMADS cells with of BIO during differentiation led to the inhibition of both differentiation programs. As shown in Fig. 5, all of the genes known to be expressed during adipocyte differentiation that we have analyzed were inhibited in the presence of BIO. Genes expressed during osteogenesis were also inhibited by BIO, although at a lower extent compared to adipogenesis-related genes. Altogether, these results are in agreement with previous reports showing that activation of Wnt or Hh pathways precludes differentiation of murine 3T3-L1 preadipose cells into adipocytes [9,21]. Our data indicate that the GSK3 inhibitor was a potent inhibitor of commitment of human preadipocyte precursors into the adipogenic lineage. The effect of Wnt pathway on osteogenesis remains more controversial. Wnt has been reported to enhance osteogenic differentiation of murine mesenchymal stem cells [22]. More recently, Cho et al. have shown that Wnt signalling suppresses osteogenic differentiation of stroma cells isolated from human adipose tissue [19]. Therefore, the effect of Wnt signalling on bone formation seems different in human versus mouse and our data with BIO-treated hMADS cells are in agreement with a negative effect of Wnt on osteogenesis of human cells.

It has been observed that patients treated with LiCl for bipolar disorder display weight gain and reduced risk of fractures, suggesting that LiCl promotes both adipogenesis and osteogenesis in humans *in vivo *[23,24]. However, a central effect of LiCl and an indirect effect on adipocytes and osteoblasts in these patients cannot be excluded.

### Differential effects of BIO treatment during early steps of adipogenesis and osteogenesis

**Figure 6:**
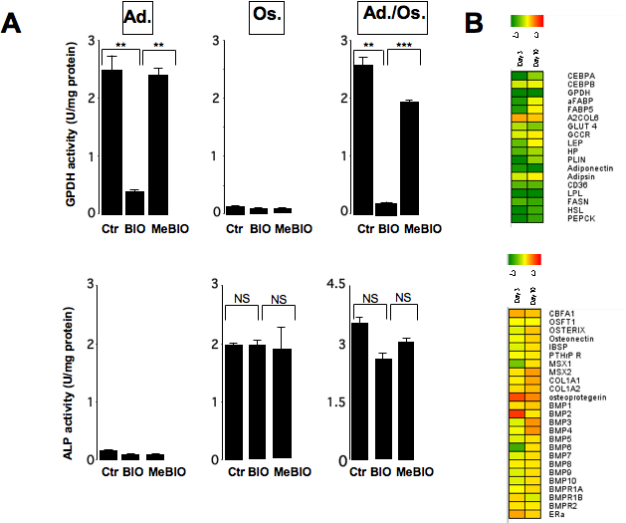
**Effects of BIO treatment during the first 3 days of differentiation on adipogenic and osteogenic abilities of hMADS cells. **hMADS3 cells were induced to undergo differentiation into the indicated lineages in the presence of BIO and MeBIO during the first 3 days. Then, compounds were withdrawn and GPDH and ALP enzymatic activities were quantified at day 10. *: p < 0.05, **: p < 0.01, NS: not significant (A). hMADS3 cells were induced to undergo differentiation into adipocytes (upper panel) or osteoblasts (lower panel) as in A) and RNAs were prepared at day ten. The expression of molecular markers was checked by qPCR (B). Results are the log2 inductions of BIO treated cells versus MeBIO treated cells. Expression data are colour-coded according to the scale that is displayed.

Then, we analyzed BIO-inhibition on adipogenic and osteogenic differentiations of hMADS cells more precisely in order to address whether a chronic treatment with the inhibitor was required. For that purpose, we treated hMADS cells during the first 3 days of differentiation and analyzed the impact of this treatment on GPDH and ALP enzymatic activities as well as on the expression of specific markers. In regards to adipogenesis, treatment of hMADS cells for the first 3 days only was sufficient to inhibit GPDH activity measured at day 10 (Fig. 6A). Analysis of adipocyte gene expression confirmed that the inhibitory effect of BIO after 3 days of treatment persisted at day 10 for most of the genes analyzed (Fig. 6B). In contrast, a treatment restricted to the first 3 days had no effect on both osteogenic markers such as ALP enzymatic activity and expression of several genes known to play a role in osteogenesis. Altogether, data indicate that treatment of hMADS cells with BIO during the early step of differentiation only led to inhibition of late steps of adipogenesis, whereas it had no effect on osteogenesis. This strongly suggests that the molecular mechanisms underlying inhibition of adipogenic and osteogenic differentiation by BIO are different.

Identification of mechanisms leading to the differential effects on adipogenesis and osteogenesis of hMADS cells could lead to a preferential use of the GSK3 inhibitors on adipocyte differentiation *in vivo*. In conclusion, our data strongly suggest that GSK3 is a promising pharmacological target to regulate both the number and differentiation of adipocyte precursors in human adipose tissue. However, we have to keep in mind that potential adverse effects could be observed due to the fact that GSK3 is implicated in numerous signalling pathways. Therefore, it would be important in the future to identify the signalling pathway mediating the adipogenic effect of GSK3.

## Conclusion

We have shown that BIO and LiCl, two inhibitors of GSK3, inhibited proliferation as well as adipogenic and osteogenic differentiations of stem cells isolated from human adipose tissue. Data demonstrate the feasibility of a pharmacological approach to regulate adipose-derived stem cell function.

## Methods

### Isolation and culture of hMADS cells

hMADS cells were obtained from the stroma of human adipose tissue as described previously [15]. Adipose tissue was collected, with the informed consent of the parents, as surgical scraps from surgical specimen of various surgeries, as approved by the Centre Hospitalier Universitaire de Nice Review Board. The cell populations that have been studied in this work were isolated from the pubic region fat pad of a 5-year old (hMADS2) and of a 4-month old (hMADS3) male donor. Proliferation medium for routine maintenance of hMADS cells is composed of DMEM (low glucose) containing 10% foetal calf serum, 10 mM HEPES, 100 U/ml penicillin and streptomycin and supplemented with FGF2 as previously reported [14]. After reaching 80% confluence, adherent cells were dissociated in 0.25% trypsin EDTA and seeded at 4500 cells/cm^2^. Cells have been maintained in low serum concentration for studying effects of GSK3 inhibitors on proliferation. This medium is composed of 60% DMEM low glucose, 40% MCDB-201, insulin (10 μg/ml), transferrine(5 μg/ml), selenium(50 ng/ml), dexamethasone (10^-9^M), ascorbic sodium acid (50 μg/ml), 2.5 ng/ml FGF2 and supplemented with 0.5% FCS. This medium containing a low serum concentration allows the maintenance of stem cell features of hMADS2 and hMADS3 cells (not shown). Human mesenchymal stromal cells isolated from bone marrow, termed hBMSC, were purchased from Cambrex and used as recommended by the manufacturer. Cultures were maintained at 37°C in a humidified gassed incubator, 5% CO_2 _in air.

### hMADS cell differentiation

Adipocyte differentiation was performed as described previously [16]. Basically, confluent cells were cultured in DMEM/Ham's F12 media supplemented with transferrin (10 μg/ml), insulin (0.86 μM), triiodothyronine (0.2 nM), dexamethasone (1 μM), isobutyl-methylxanthine (100 μM) and rosiglitazone (500 nM). Three days later, the medium was changed (dexamethasone and isobutyl-methylxanthine were omitted). Neutral lipid accumulation was assessed by Oil red O staining [25]. For osteoblasts, confluent cells were cultured in α-MEM medium containing 10% FCS, L-ascorbic acid phosphate (50 μg/ml), β-glycerophosphate (10 mM) and 100 nM dexamethasone. Alizarin red staining was performed as previously described [25]. For simultaneous adipocyte and osteoblast differentiations, confluent cells were cultured in the differentiation medium consisting in 50% adipogenic and 50% osteogenic media.

### Measurement of adipogenic and osteogenic specific enzymatic activities

Cell homogenates for both Glycerol-3-phosphate dehydrogenase (GPDH) and Alkaline phosphatase (ALP) enzymatic activity measurements were prepared in 20 mM Tris-HCl pH 7.5 buffer containing 1 mM EDTA and 1 mM 2-mercaptoethanol. GPDH activity was measured by the absorbance at 340 nm as described previously [26] and ALP activity was assayed using the p-Nitrophenyl Phosphate Liquid Substrate System (Sigma). Absorbance was measured at 412 nm.

### Cell proliferation assays

Cells were plated onto 12-well plates (10^4 ^cells per well). GSK3 inhibitors were added 24 hours after cell plating in order to avoid a potential effect of the compounds on the efficiency in cell attachment. After the appropriate time, cells were trypsinized as mentioned above and counted with a Coulter counter. For each experiment, three wells per condition were counted.

### Clonal assays

Cells were plated at a density of 10 cells/cm^2 ^in 100-mm^2 ^dishes. 15 days after plating cells were fixed with 0.25% glutaraldehyde and stained with 0.1% crystal violet. Colonies containing at least 40 cells were enumerated under a light microscope. Medium was changed 3 times a week.

### Immunocytochemistry

hMADS cells were rinsed twice with PBS at 4°C, and fixed with 4% paraformaldehyde in PBS for 15 min at room temperature. Free aldehydes were quenched by a 15-min incubation with 1 M glycine in PBS. Fixed cells were permeabilized with 0.5% Triton for 10 min, and unspecific reactions blocked with 5% bovine serum albumin in PBS for 30 min. Cells were then incubated for 1 h with an anti β-catenin (Santa Cruz Biotechnology) antibody diluted in 5% bovine serum albumin in PBS, followed by a 488-Alexa Fluor conjugated secondary antibody. Nuclei were counterstained with DAPI. Images were taken on an LSM510 META confocal microscope (Zeiss).

### RT-PCR analysis

Total RNA was extracted using TRI-Reagent™ kit (Euromedex, France) according to the manufacturer's instructions and RT-PCR analysis was conducted as described previously [14]. All primers sequences, designed using Primer Express software (Applied Biosystems, France), see Additional file 4. For quantitative PCR, final reaction volume was 25 μl, including specific primers (0.4 μM), 5 ng of reverse transcribed RNA and 12.5 μl SYBR green master mix (Applied Biosystems, France). Quantitative PCR conditions were as follows: 2 min at 50°C, 10 min at 95°C, followed by 40 cycles of 15 sec at 95°C, 1 min at 60°C. Real-time PCR assays were run on an ABI Prism 7000 real-time PCR machine (Applied Biosystems, France). Normalization was performed using the geometrical average of the housekeeping genes G6PDH, POLR2A and TBP. Quantification was performed using the comparative-ΔCt method. Level of expression was represented using the genesis software [27]. This software generates expression images using a colour code according to the expression intensities.

### Statistical analysis

Statistical significance was checked by using t-tests whenever comparing two conditions or a one-way ANOVA followed by t-tests whenever comparing more than two conditions. Asterisks indicate the significance levels as mentioned in the figure legends.

## Authors' contributions

LEZ performed most of the experiments, participated in their design and helped to draft the manuscript. BW carried out cellular studies. PV, CF and PP carried out some molecular studies. PP participated also in the design of the study. CD conceived of the study, coordination and drafted the manuscript. All authors read and approved the final manuscript.

## Supplementary Material

Additional File 1 **Activation of β-catenin pathway by BIO**. In order to evaluate the activation of the β-catenin pathway, hMADS cells were nucleofected as described in Zaragosi et al. [14] with a plasmid carrying firefly luciferase under the control of a Tcf/Lef response element (TopLuc). An inactive version of the response element was used as a control (FopLuc). Twenty four hours after nucleofection, cells were treated with BIO and 24 h after BIO treatment, cells were analyzed for luciferase expression. Renilla luciferase was co-nucleofected and used for normalization. Data indicated that β-catenin pathway was activated in hMADS cells after inhibiting GSK3. Click here for file

Additional File 2 **Morphology of hMADS3 cells after treatment with GSK3 inhibitors**. Cells were maintained in medium supplemented with 0.5% FCS in the absence (Control) or presence of 0.5 μM BIO or 20 mM LiCl for 5 days. Click here for file

Additional File 3 **Impact on differentiation of GSK3 inhibition during cell proliferation**. hMADS cells were maintained in the absence or presence of BIO or MeBio for 5 days. Then, cells were collected and plated at high cell density without any GSK3 inhibitor. Two days after cells reached confluence and were induced to undergo differentiation into adipocytes. GPDH activity was quantified seven days after induction of differentiation. Click here for file

Additional File 4 **Primer sequences used for quantitative PCR**. Description: Primers sequences were designed using Primer Express software (Applied Biosystems, France). Click here for file
