# Supplementary material for: Effects of GSK3 inhibitors on in vitro expansion and differentiation of human adipose-derived stem cells into adipocytes
Source: BMC Cell Biol. 2008 Feb 13;9:11. doi: 10.1186/1471-2121-9-11 (PMC2257931; doi:10.1186/1471-2121-9-11)
Supplement: Additional File 1 — Activation of β-catenin pathway by BIO. In order to evaluate the activation of the β-catenin pathway, hMADS cells were nucleofected as described in Zaragosi et al. [14] with a plasmid carrying firefly luciferase under the control of a Tcf/Lef response element (TopLuc). An inactive version of the response element was used as a control (FopLuc). Twenty four hours after nucleofection, cells were treated with BIO and 24 h after BIO treatment, cells were analyzed for luciferase expression. Renilla luciferase was co-nucleofected and used for normalization. Data indicated that β-catenin pathway was activated in hMADS cells after inhibiting GSK3. [file 1471-2121-9-11-S1.PDF]

	treatment	fold induction
FopLuc	control	1.00
	BIO 2.5 μM	1.01
TopLuc	control	1.00
	BIO 2.5 μM	17.85
