# Supplementary figures and images for: Effects of GSK3 inhibitors on in vitro expansion and differentiation of human adipose-derived stem cells into adipocytes
Source: BMC Cell Biol. 2008 Feb 13;9:11. doi: 10.1186/1471-2121-9-11 (PMC2257931; doi:10.1186/1471-2121-9-11)

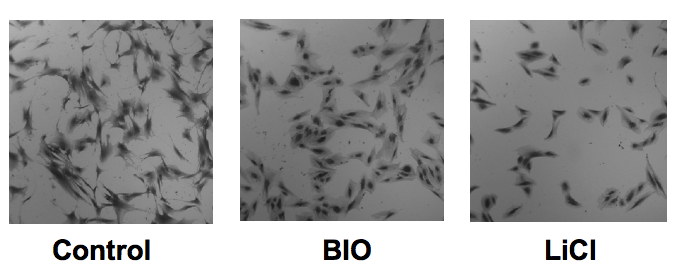

Supplement: Additional File 2 — Morphology of hMADS3 cells after treatment with GSK3 inhibitors. Cells were maintained in medium supplemented with 0.5% FCS in the absence (Control) or presence of 0.5 μM BIO or 20 mM LiCl for 5 days. [file 1471-2121-9-11-S2.TIFF]

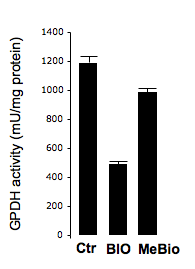

Supplement: Additional File 3 — Impact on differentiation of GSK3 inhibition during cell proliferation. hMADS cells were maintained in the absence or presence of BIO or MeBio for 5 days. Then, cells were collected and plated at high cell density without any GSK3 inhibitor. Two days after cells reached confluence and were induced to undergo differentiation into adipocytes. GPDH activity was quantified seven days after induction of differentiation. [file 1471-2121-9-11-S3.TIFF]
