# Supplementary material for: Effects of GSK3 inhibitors on in vitro expansion and differentiation of human adipose-derived stem cells into adipocytes
Source: BMC Cell Biol. 2008 Feb 13;9:11. doi: 10.1186/1471-2121-9-11 (PMC2257931; doi:10.1186/1471-2121-9-11)
Supplement: Additional File 4 — Primer sequences used for quantitative PCR. Description: Primers sequences were designed using Primer Express software (Applied Biosystems, France). [file 1471-2121-9-11-S4.PDF]

Gene	Accession number	Sense primer	Anti-sense primer
**CEBPA**	NM_004364	CTTGTGCCTTGGAAATGCAA	GCTGTAGCCTCGGGAAGGA
**CEBPB**	NM_005194	AACCAACCGCACATGCAGAT	GGCAGAGGGAGAAGCAGAGAGT
**PPARa**	NM_005036	GGCGAACGATTCGACTCAAG	TCCAAAACGAATCGCGTTGT
**PPARd**	NM_177435	AGGCTATCCAGGACACCATCCT	CGTGCTCGGTGACCAGTTG
**PPARγ**	NM_005037	AGCCTCATGAAGAGCCTTCCA	TCCGGAAGAAACCCTTGCA
**RXRa**	NM_002957	GCCCTCGAGCCAATGAGAA	GGAGTCGGGAGTCTGAAACCA
**FXR**	NM_005123.1	TTTGACGGAAATGGCAACCA	TTCAACCGCAGACCCTTTCA
**GPDH**	NM_005276	TTGTGGTGCCCCATCAGTTC	CCCAATCACTTCCGAGATGA
**aFABP**	NM_001442	TGTGCAGAAATGGGATGGAAA	CAACGTCCCTTGGCTTATGCT
**FABP5**	NM_001444	ACAGCTGATGGCAGAAAAACTCA	ACACTCCACCACTAATTTCCCATCT
**A2COL6**	NM_058175	GGTCATCTCGCCGGACACTA	ACGAACTGCTTCATGTGGAAGAG
**GLUT4**	NM_001042	CGTCGGGCTTCCAACAGATA	CACCGCAGAGAACACAGCAA
**GCCR**	NM_000176	AAAGAGACGAATGAGAGTCCTTGGA	GCTTGCAGTCCTCATTCGAGTTT
**LEP**	NM_000230	AGGGAGACCGAGCGCTTTC	TGCATCTCCACACACCAAACC
**HP**	NM_005143	CAGCACAGTCCCCGAAAAGA	AGGTCGTGAACGGCAAAGG
**PLIN**	NM_002666	ACCCCCCTGAAAAGATTGCTT	GATGGGAACGCTGATGCTGTT
**ACDC**	NM_004797	GCAGTCTGTGGTTCTGATTCCATAC	GCCCTTGAGTCGTGGTTTCC
**Adipsin**	NM_001928	AGGGTCACCCAAGCAACAAAG	TACGTGGCCCATGCTGATCT
**CD36**	NM_000072	GGGAAAGTCACTGCGACATGAT	ACGTCGGATTCAAATACAGCATAGA
**LPL**	NM_000237	TGGAGGTACTTTTCAGCCAGGAT	TCGTGGGAGCACTTCACTAGCT
**FASN**	NM_004104	TGAACTCCTTGGCGGAAGAGA	GTAGGACCCCGTGGAATGTCA
**HSL**	NM_005357	GCACTACAAACGCAACGAGACA	GGTTCTGTGTGATCCGCTCAA
**PEPCK**	NM_002591	AGGGCCATCAACCCAGAAAA	TGGTCTCGGCCACATTGG
**CBFA1**	NM_004348	AGTGGACGAGGCAAGAGTTTCA	GGGTTCCCGAGGTCCATCTA
**OSTF1**	NM_012383	ACCAGCAGAACAAGTTGGGAGATA	AGCACCTTTTGCCAGAAGCA
**OSTERIX**	NM_152860	GGCAGCGTGCAGCAAATT	CATCCCCCATGGTTTTGGA
**Osteonectin**	NM_003118	CCCATTGGCGAGTTTGAGAA	TTGCAAGGCCCGATGTAGTC
**ISBP**	NM_004967	CTGCCTTGAGCCTGCTTCCT	AGCACAGGCCATTCCCAAA
**PTHrPR**	NM_002820	GAACTCGCTCTGCCTGGTTAGA	GAGGCTACGGGCCAGAGAAG
**MSX1**	NM_002448	AACCCTCACACTGCTCCAGTTTC	TGCCCTCAGTTTCCCCATCT
**MSX2**	NM_002449	AGGAACCCGGCCGATATTC	CGAGGAGCTGGGATGTGGTA
**Osteoprotegerin**	NM_002546	CCTCCAAGCCCCTGAGGTT	CCTGGGTGGTCCACTTAATGG

**BMP1**	NM_006129	AGCAGCAATTGGGTTGGAAAG	GGCGATTGAATGTGGCCATA
**BMP2**	NM_001200	TGTGGACGCTCTTTCAATGGA	GGGAAGCAGCAACGCTAGAAG
**BMP4**	NM_001202	CCTGTTGTGTGCCCACTGAAC	ATCTCAGCGGCACCCACAT
**BMP6**	NM_001718	GCGATTGTGCAGACCTTGGT	CCGAGATGGCATTTAGCTTAGTTG
**BMPR1A**	NM_004329	GTGGGCACCAAACGCTACA	TTCCACGATCCCTCCTGTGA
**BMPR1B**	NM_001203	CCTCCCTCTGCTGGTCCAA	CTTTTCGCCACGCCACTTT
**BMPR2**	NM_001204	AAACCTGCAATTTCCCATCGA	GGCGCACCAGTCTATTTCCA
**TWIST1**	NM_000474	GCCGGAGACCTAGATGTCATTG	CCACGCCCTGTTTCTTTGAA
**G6PDH**	NM_004285	ACCTTCGCAGCCGTCCTAGT	ATCCGAGCGTAGCCCACTCT
**POLR2A**	NM_000937	TGGGTGTGCCCCGACTTAA	TCCAGACGGCACAGAATATCCT
**TBP**	NM_003194	ACGCCAGCTTCGGAGAGTTC	CAAACCGCTTGGGATTATATTCG
